# Enzymatic and non-enzymatic removal of organic micropollutants with spent mushroom substrate of *Agaricus bisporus*

**DOI:** 10.1007/s00253-024-13132-3

**Published:** 2024-04-19

**Authors:** Brigit van Brenk, Fleur E.L. Kleijburg, Antoine J.B. Kemperman, Walter G.J. van der Meer, Han A.B. Wösten

**Affiliations:** 1 Microbiology, Department of Biology, Utrecht University, Padualaan 8, Utrecht, 3584 CH the Netherlands; 2 Membrane Science and Technology cluster, University of Twente, P.O. Box 217, Enschede, 7500 AE the Netherlands; 3 Oasen, PO BOX 122, Gouda, 2800 AC the Netherlands

**Keywords:** Organic micropollutants, Bioremediation, Water purification, Fungus, Spent mushroom substrate, *Agaricus bisporus*

## Abstract

Water bodies are increasingly contaminated with a diversity of organic micropollutants (OMPs). This impacts the quality of ecosystems due to their recalcitrant nature. In this study, we assessed the removal of OMPs by spent mushroom substrate (SMS) of the white button mushroom (*Agaricus bisporus*) and by its aqueous tea extract. Removal of acesulfame K, antipyrine, bentazon, caffeine, carbamazepine, chloridazon, clofibric acid, and *N, N*-diethyl-*meta*-toluamide (DEET) by SMS and its tea was between 10 and 90% and 0–26%, respectively, in a 7-day period. Sorption to SMS particles was between 0 and 29%, which can thus not explain the removal difference between SMS and its tea, the latter lacking these particles. Carbamazepine was removed most efficiently by both SMS and its tea. Removal of OMPs (except caffeine) by SMS tea was not affected by heat treatment. By contrast, heat-treatment of SMS reduced OMP removal to < 10% except for carbamazepine with a removal of 90%. These results indicate that OMP removal by SMS and its tea is mediated by both enzymatic and non-enzymatic activities. The presence of copper, manganese, and iron (0.03, 0.88, and 0.33 µg L^-1^, respectively) as well as H_2_O_2_ (1.5 µM) in SMS tea indicated that the Fenton reaction represents (part of) the non-enzymatic activity. Indeed, the in vitro reconstituted Fenton reaction removed OMPs > 50% better than the teas. From these data it is concluded that spent mushroom substrate of the white button mushroom, which is widely available as a waste-stream, can be used to purify water from OMPs.

**Supplementary Information:**

The online version contains supplementary material available at 10.1007/s00253-024-13132-3.

## Key points

• *A. bisporus SMS effectively removes OMPs from water, outperforming its tea.*

• *A. bisporus SMS utilizes enzymatic and non-enzymatic activities for OMP removal.*

• *A. bisporus SMS is a promising bioremediation substrate for OMP removal.*

**Supplementary Information:**

The online version contains supplementary material available at 10.1007/s00253-024-13132-3.

## Introduction

Levels of organic micropollutants (OMPs) are increasing in surface- and groundwater, including the water bodies that are used as sources for drinking water (Schaible 2020; Wettenbank Overheid 2017; 2021; WHO 2022; UNEP 2017). These compounds are currently being removed by for example active carbon or membrane filtration (Crittenden et al. 2012). However, active carbon filters do not remove all OMPs and need regeneration, while use of membranes produces a waste stream with concentrated levels of these compounds (Crittenden et al. 2012; Virkutyte et al. 2010). This waste water stream is transferred to waste water treatment plants (WWTPs) or is even directly discharged to surface water. After treatment by the WWTP, part of the OMPs still end up in the surface water due to inefficient elimination (Virkutyte et al. 2010). This continuous re-pollution of surface waters results in increased concentrations of OMPs, thereby enhancing the risk of toxic effects on living (aquatic) organisms (Bartram et al. 2002; Kortenkamp et al. 2011).

Due to the high variety of OMPs, it is challenging to design a system that removes all these compounds. Yet, wood- and litter-degrading fungi may have such potential. These fungi are considered the recyclers in nature due to their ability to mineralize lignocellulose (Blanchette 1995; Worrall et al. 1997). These microbes use non-specific oxidative enzymes to degrade lignin, which can also be used to degrade aromatic molecules such as dyes, pharmaceuticals and herbicides (Barr and Aust 1994). *Agaricus bisporus* produces these ligninolytic enzymes when grown on compost during commercial white button mushroom production (Colmenares-Cruz et al. 2017). The spent mushroom substrate (SMS), a waste stream after harvesting of the mushroom (Gerrits 1994), still contains ligninolytic activity (Jordan et al. 2008; McGee 2018). SMS of *A. bisporus* can be used to bioremediate dye-containing water (van Brenk 2024). Also, SMS of several edible fungi have been tested for their ability to remove or sorb heavy metals, poly-aromatic hydrocarbons, pesticides, pharmaceuticals and other OMPs in soil (Leong et al. 2022; Phan and Sabaratnam 2012) and waste water (García-Delgado et al. 2017; Hultberg et al. 2020; Toptas et al. 2014). Adding SMS to a heavy metal-contaminated soil can change chemical properties of the soil, which can reduce the mobility of metals. This so-called phytostabilization results in the conversion of soluble heavy metals into insoluble compounds, for example by binding to organic matter (Wei et al. 2020; Yu et al. 2021). In addition, SMS of different mushroom forming fungi can degrade OMPs (Law et al. 2003; Purnomo et al. 2010; Sadiq et al. 2018; Zang et al. 2020) or stimulate OMP degrading bacteria in the soil (García-Delgado et al. 2015; Wang et al. 2016). In the case of waste water, SMS of *A. bisporus* was shown to reduce contamination by sorbing dyes and heavy metals (García-Delgado et al. 2017; Toptas et al. 2014). A higher pH of the waste water resulted in a higher adsorption. Furthermore, an extracted tea of SMS of *Pleurotus ostreatus* removed over 80% of diclofenac from waste water (Hultberg et al. 2020). Overall, the use of SMS to remove OMPs is promising. Yet, the degrading mechanisms are poorly described (Ghose and Mitra 2022).

In this study, the potential of *A. bisporus* SMS was assessed to remove a mixture of eight pharmaceuticals and herbicides. It is shown that SMS has a higher removal activity than its tea (i.e. its aqueous extract) and that both enzymatic and non-enzymatic activity are responsible for OMP removal that can be as high as 90% after a 2-day incubation.

## Materials and methods

### Strains and culture conditions

Boxes (26 × 20 × 20 cm; Manutan, www.manutan.nl) were filled with 2.5 kg horse manure-based compost that was colonized with *A. bisporus* strain A15 (Sylvan, www.sylvaninc.com) (so-called PIII compost; CNC Grondstoffen, www.cncgrondstoffen.nl) and topped with 1 kg casing soil (CNC Grondstoffen) (Herman et al. 2020). After 14 days of growth at 22 °C and 80% relative humidity (RH), mushroom formation was induced by adding 100 mL water and venting at 18 °C and 80% RH. The first and second flush were harvested 9–12 and 21–23 days after venting, respectively. The resulting SMS was homogenized (broken and mixed by hand) and stored at -20 °C. SMS tea was made by mixing SMS with demi water (1 g per 20 mL), shaking for 1 h at 175 rpm at room temperature (21 °C), and removing insoluble SMS particles by filtering through a Melitta^®^ coffee filter (www.melitta-group.com). SMS teas were used directly or stored at -20 °C.

### Treatment of OMPs by SMS and SMS tea

OMPs (0.1–1 mg L^-1^; all from Sigma Aldrich, www.sigmaaldrich.com) (Table 1) were mixed with SMS (1 g per 20 mL OMP solution) in a 250 mL Erlenmeyer with a screw lit. Triplicates were incubated at room temperature at 100 rpm for up to 7 days. Similarly, 20 mL OMP mix (0.1–1 mg L^-1^) was added to 180 mL SMS tea (18 mL per g SMS) in a 250 mL Erlenmeyer with screw lit. Triplicates were incubated at room temperature at 100 rpm for up to 7 days. Water without OMPs added to tea or SMS and OMP mixtures without tea or SMS served as controls.

**Table 1:** Organic micropollutants used in this study including the concentrations of these compounds used in the experiments to study their removal over time (^a^), upon heat treatment of SMS or SMS tea (^b^), or in fenton-like reactions (^c^)

Compound	CAS No.	m/z	ESI^−/+^	Concentration (mg L^− 1^)
Acesulfame K	55589-62-3	161.85	-	0.1^a^ or 1^b, c^
Antipyrine	60-80-0	189.00	+	0.1^a^ or 1^b, c^
Bentazon	25057-89-0	238.95	-	0.1^a^ or 1^b, c^
Caffeine	58-08-2	195.00	+	0.1^a^ or 1^b, c^
Carbamazepine	298-46-4	236.95	+	0.1^a^ or 1^b, c^
Chloridazon	1689-60-8	221.90	+	0.1^a^ or 1^b, c^
Clofibric acid	882-09-7	212.95	-	0.1^a^ or 1^b, c^
DEET	134-62-3	192.05	+	0.1^a, c^ or 1^b^

Methanol extraction was done to determine OMP adsorption to insoluble SMS particles. To this end, the insolubles were separated from the aqueous solution with a strainer, followed by addition of methanol (4 mL per g SMS). Samples were incubated for 20 min and / or overnight at room temperature at 100 rpm. Water without OMPs added to SMS and OMP mixtures without SMS served as controls.

### Analysis of OMP removal

Samples were filtered using 0.22 μm centrifuge tubes (Corning^®^ Costar^®^ spin-X^®^, CLS8161, www.sigmaaldrich.com) before 50 µL samples were analyzed by LC-MS (HPLC e2695 plus Autosampler, ACQUITY QDa Mass Detector, Waters Corporation, www.waters.com). Separation of molecules was done using 0.01% formic acid in ultrapure water (solvent A; Sigma-Aldrich) and 0.01% formic acid in acetonitrile (solvent B; Sigma-Aldrich) at 35 °C at a flow of 0.8 mL min^-1^. A linear gradient from 95% solvent A: 5% solvent B to 45% solvent A: 55% solvent B was obtained in 10 min. After flowing the latter solution for 2 min, the column (XBridge^®^ C18 3.5 μm, 100 × 4,6 mm; Waters Corporation) was eluted with 20% solvent A: 80% solvent B for 1 min. Compounds were quantified based on calibration curves (1–1000 ug L^-1^) using single ion recording (SIR; Tabel 1) with a probe temperature of 600 °C and positive and negative capillary of 0.3 kV.

Percentage removal was calculated by comparing day 0 with day z (Eq. 1).

1 $$percentage\,OMP\,removal\, = \,\frac{{{D_0} - {D_z}}}{{{D_0}}} \times 100\%$$

Where *D*_0_ is the concentration measured at day 0 and *D*_*z*_ the concentration measured at a certain day.

Percentage converted was measured by comparing the concentration removal with the concentration measured after methanol extraction (Eq. 2).

2 $$percentage\,OMP\,converted\, = \,\frac{{\left( {{D_0} - {D_z}} \right) - S{D_z}}}{{({D_0} - {D_z})}} \times 100\%$$

Where *D*_0_ is the concentration measured at day 0 and *D*_*z*_ the concentration measured at a certain day with *SD*_*z*_ the concentration measured after the methanol extraction.

### Oxidative enzyme activity, hydrogen peroxide availability and biological viability

To determine oxidative enzyme activity, a 50 µL sample was mixed with 150 µL 1 mM 2,2′-azino-bis(3-ethylbenzothiazoline-6-sulfonic acid) diammonium salt (ABTS) in citric phosphate buffer, pH 4, in a 96 wells plate (Greiner Bio-One, Cellstar 655180, www.gbo.com). Absorption at 420 nm was measured every min for 30 min with a spectrophotometer (Biotek Synergy HT Reader, Agilent, www.agilent.com). Activity (mM s^-1^ L^-1^) was calculated using the law of Lambert-Beer with an extinction coefficient of 36,000 M^-1^ cm^-1^.

H_2_O_2_ concentration was measured in 5 replicates of SMS and its tea using a hydrogen peroxide assay kit (Amplex™ Red Hydrogen Peroxide/Peroxidase Assay Kit, A22188, ThermoFisher Scientific Inc, www.thermofisher.com). A spectrophotometer (Biotek Synergy HT Reader, Agilent) was used to measure the fluorescence (Em = 530/25, Ex = 590/35) and concentration was calculated using a calibration curve (0.3–5 µM) based on aqueous H_2_O_2_ solutions (Sigma-Aldrich). These solutions were also used to determine the stability of H_2_O_2_ over time.

Viability of microorganisms in SMS tea was tested by inoculating dilution series on LB medium plates. Microorganisms were grown for one week at 25 °C .

### Quantification of inorganic compounds

SMS tea was filtered through a 0.2 μm filter (Filtropur S microfilter, Sarstedt Inc., www.sarstedt.com) and diluted 10 times with demi water. The Berthelot reaction in a continuous flow analyzer (SAN + + System Continuous Flow Analyzer, Skalar, www.skalar.com) was used to determine the concentration of NH_4_^+^. The system sample and wash times were set at 80 s. A dilution series of an aqueous NH_4_^+^ solution was used as to make a calibration curve (0.07–5 mM; Sigma-Aldrich). The amount of NH_4_^+^ in SMS tea was calculated with a linear regression model subtracting the amount in the demi water blank. The concentration of Ca^2+^, Cu^2+^, Fe^3+^, K^+^, Mg^2+^, Mn^2+^, MoO_4_^2-^, Na^+^, PO_4_^3-^, SO_4_^2-^, and Zn^2+^ was determined on a Inductively Coupled Plasma Optical Emission Spectrometer (iCAP 6000 Series, ThermoFischer Scientific Inc). To this end, the diluted SMS tea was acidified with nitric acid (1% v/v). The counts per second of Ca^2+^, Cu^2+^, Fe^3+^, K^+^, Mg^2+^, Mn^2+^, MoO_4_^2-^, Na^+^, PO_4_^3-^, SO_4_^2-^, and Zn^2+^ were recorded at 422.673 nm, 327.396 nm, 238.204 nm, 766.490 nm, 279.079 nm, 260.569 nm, 204.589 nm, 818.326 nm, 214.914 nm, 182.034 nm, and 206.200 nm, respectively. The concentrations of these ions were calculated with linear regression models based on calibration curves (0.01 µM − 7.5 mM, compound depending; Sigma-Aldrich). Correction was done by subtracting the counts per second for a demi water blank. All measurements were performed using triplicates.

### OMP removal by fenton reaction compared to SMS tea and sterilized SMS tea

SMS tea (15 mL per 1 g of SMS), either or not heat treated (121 °C for 20 min) and filtered through a 0.2 μm filter (Filtropur S microfilter, Sarstedt Inc.), was diluted by adding each OMP (i.e. not a mixture of these molecules) (1 mg L^-1^, except for 100 µg L^-1^*N, N*-diethyl-*meta*-toluamide (DEET) and dH_2_O or H_2_O_2_ (0.97µM). For the Fenton reaction, CuSO_4_ (2.42 mM), MnSO_4_ (45.21 mM), FeSO_4_ (13.45 mM), and H_2_O_2_ (0.97 µM) were mixed with 1 mg L^-1^ OMP (each individually; not mixed), except for DEET, that had a start concentration of 100 µg L^-1^. The metal concentrations in the reaction mixture were the same as found in SMS tea. As a control, the same conditions were used in the absence of the OMPs. Removal of the OMPs was assessed by LCMS (see above). All conditions were performed using triplicates.

### Statistics

Statistics was done in R (RStudio Version 4.2.3, 2023 RStudio, Inc., www.posit.co) using a *p*-value ≤ 0.05. One-way Anova with Tukey’s HSD post-hoc test was used to determine significant differences in concentrations.

## Results

**Fig. 1:**
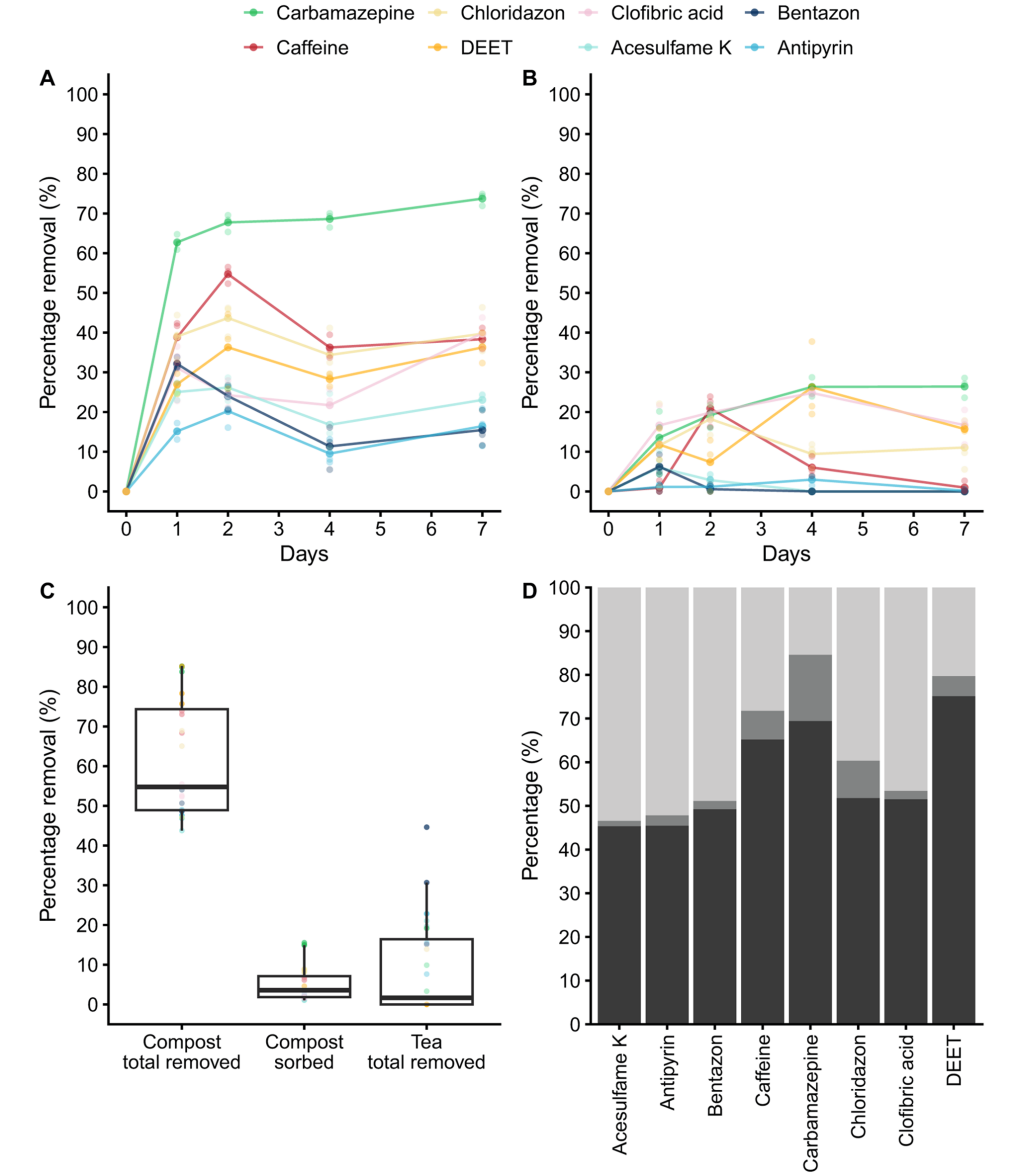
Removal (converted and sorbed) of eight different organic micropollutants in time by SMS (**A**) and SMS tea (only converted) (**B**). SMS removed OMPs better than the tea with a sorption ≤ 15% after 2 days of incubation with SMS (**C**). This is shown in more detail in (**D**) where percentage converted OMPs are indicated by dark grey shading, percentage sorbed OMPs by grey shading, and percentage OMPs that are still in solution by light grey shading. ^a,b,c^ indicates significant differences *p* < 0.05

An aqueous solution of a mixture of acesulfame K, antipyrine, bentazon, caffeine, carbamazepine, chloridazon, clofibric acid, and DEET was incubated with SMS. Sorption (% mass per volume) of these compounds to SMS particles was between 0 and 15% (Fig. 1C,D) and 0–29% (data not shown) after overnight and a 20 min incubation period, respectively, and was not affected by heat treatment (data not shown). Only bentazon and carbamazepine sorbed significantly better after the 20 min incubation when compared to overnight incubation (i.e. 9% vs. 3% and 29% vs. 15%). As a control, sorption to phase II compost (substrate without *A. bisporus*) was assessed. Sorption was similar to SMS for the OMPs except acesulfame K, caffeine and DEET that showed higher sorption to the PII compost (Supplemental Fig. S1).

Removal (i.e. the sum of conversion and sorption) ranged between 15% and 73% after seven days of incubation (Fig. 1A). Antipyrine (20%) and carbamazepine (68%) were removed the least and most efficiently after two days, respectively (Fig. 1A). The highest removal rate was observed within the first 24 h, while maximal removal was found after 2 days of incubation in the case of 7 out of 8 compounds. Only removal of carbamazepine was significantly higher at later time points. A total of 11–26% of carbamazepine, chloridazon, clofibric acid and DEET was removed (only conversion) by SMS tea (Fig. 1B), while removal of the other OMPs was < 10%. Of the OMPs, carbamazepine was removed most efficiently (26%). Together, SMS was superior in removal of OMPs compared to SMS tea (Fig. 1C).

**Fig. 2:**
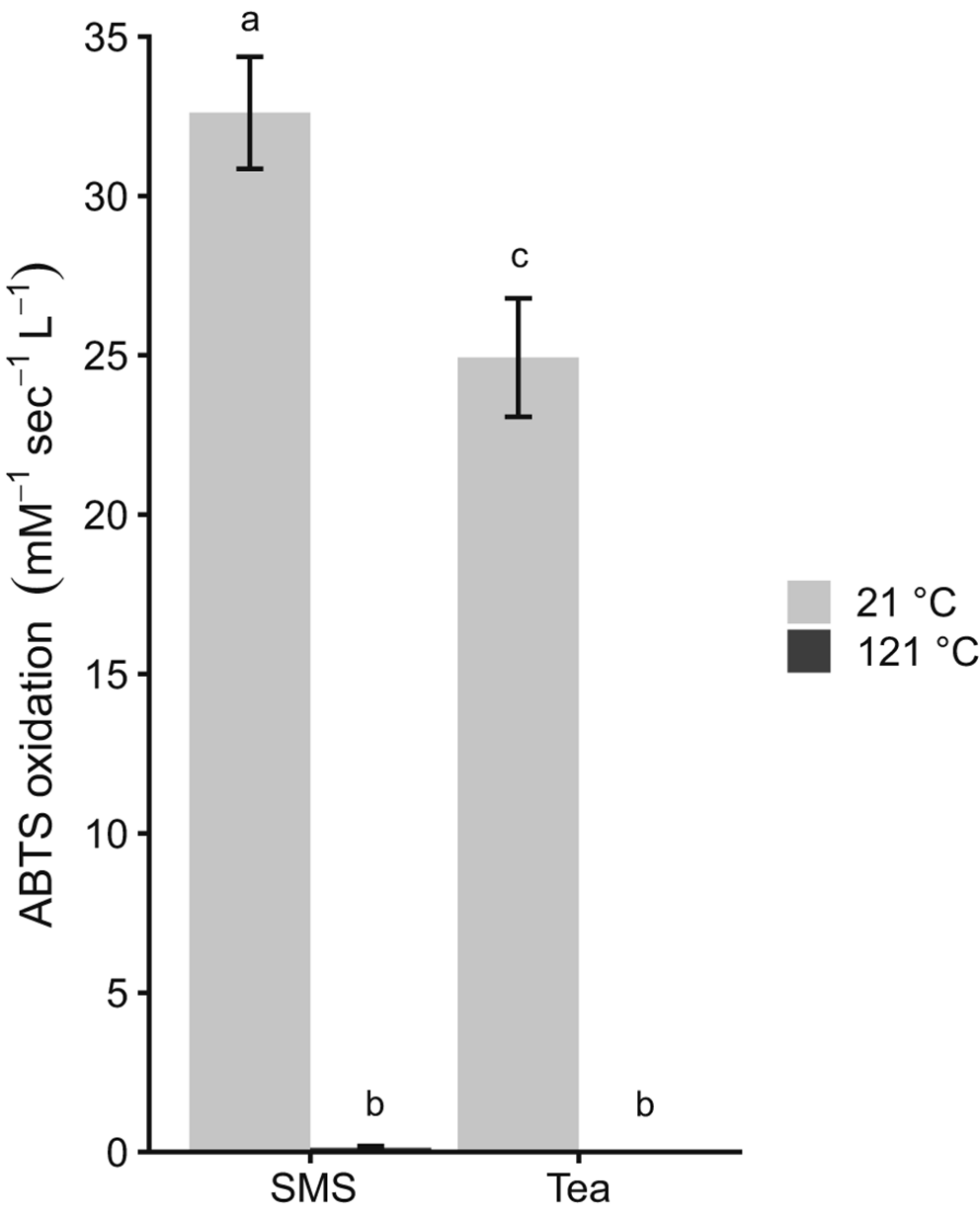
ABTS oxidation by SMS and its tea after treatment at 21–121 °C. SMS treated at 21 °C had a higher activity than tea treated at this temperature. ABTS activity was abolished for both SMS and tea after treatment at 121 °C. ^a,b,c^ indicates significant differences *p* < 0.05

**Fig. 3:**
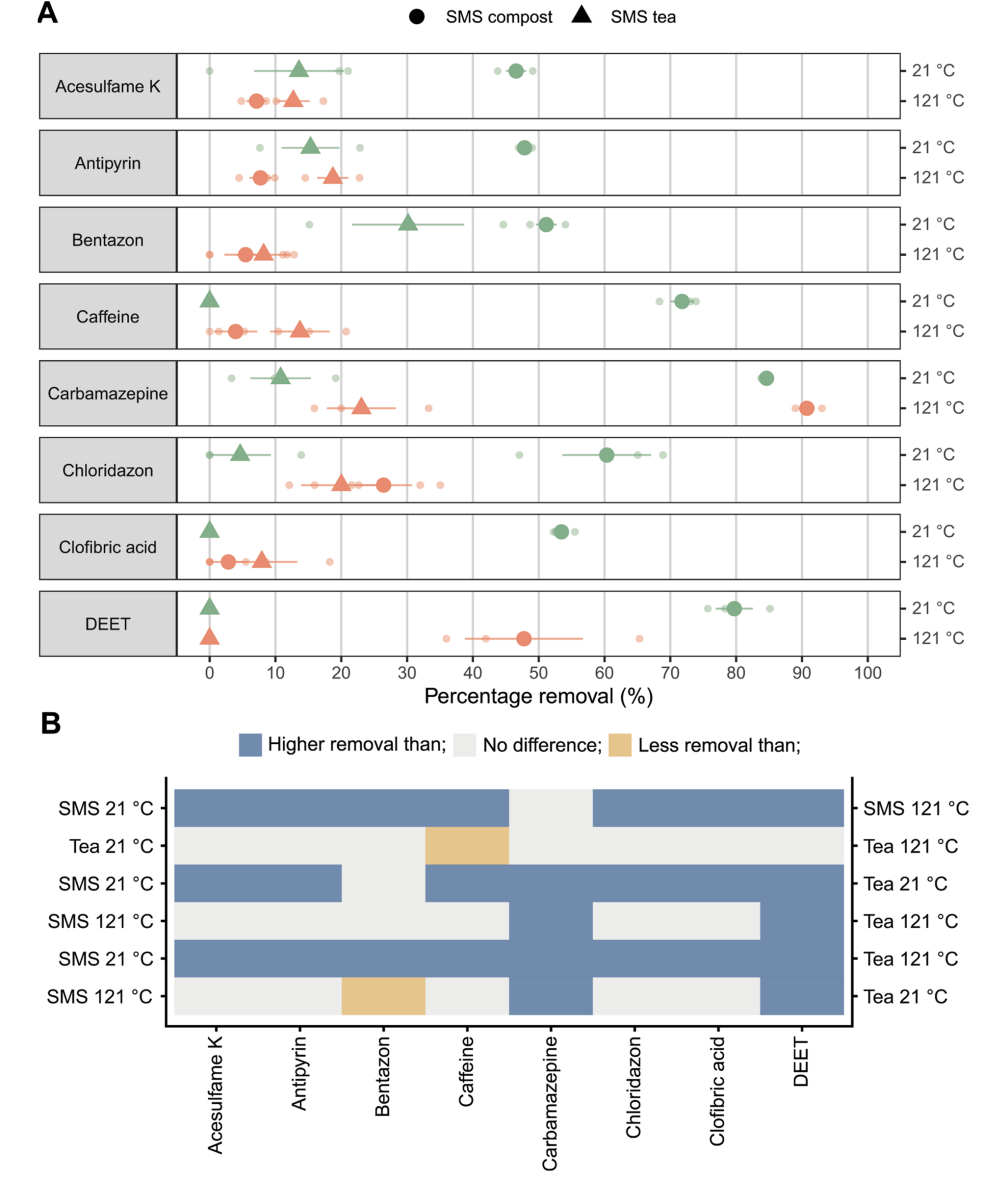
Removal of OMPs by non- or heat-treated SMS and its tea (**A**). Overall, heat-treatment had a negative effect on OMP removal by SMS, but not in the case of its tea (**B**). For instance, removal of OMPs (indicated in the x-axis) was higher (*p* < 0.05) in the case of untreated SMS (left y-axis) when compared to heat-treated SMS (right y-axis), indicated by the blue shading in the upper row. Removal was not affected when SMS tea (left y-axis) had been heat-treated (right y-axis), indicated by the grey shading. Caffeine was even better removed by heat-treated tea (right y-axis) when compared to untreated tea (left y-axis), indicated by the yellow shading in the second row from the top

SMS and its tea were treated at 121 °C during 20 min to kill microbes and their (secreted) enzymes. As expected, the heat-treated samples did not show oxidative enzyme activity as revealed by an ABTS assay (Fig. 2) and no bacterial growth was observed on LB plates (data not shown). Heat-treated SMS showed < 10% removal of OMPs, except for carbamazepine, chloridazon, and DEET (Fig. 3A). In the latter cases, 90%, 26%, and 47% were still removed, respectively, compared to 84%, 60%, and 79% in the case of the untreated samples. Heat treatment of SMS tea did not result in reduced OMP removal. In fact, caffeine even showed a higher removal after the treatment (Fig. 3B). Together, heat treated SMS and its untreated tea showed similar OMP removal except for carbamazepine and DEET that were better removed by SMS. Remarkably, removal of bentazon by heat-treated SMS was lower than the untreated tea, while for all the other compounds no differences or more removal was found. Together, results imply that OMP removal in SMS and its tea is mediated by enzymatic and non-enzymatic activities. The former is higher in SMS, while the latter seems to play a major role in its tea.

**Fig. 4:**
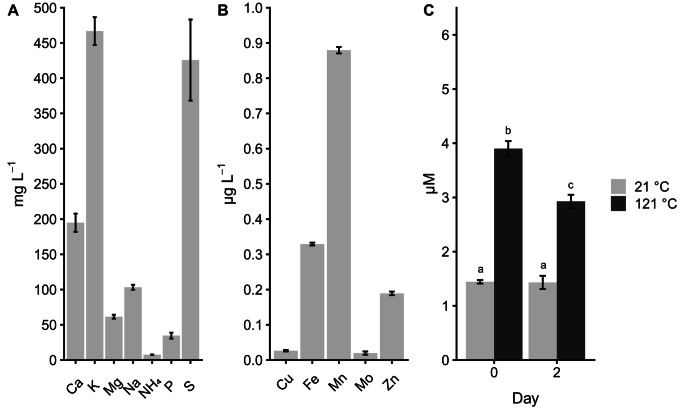
SMS tea nutrient composition (**A** & **B**) and concentration of H_2_O_2_ at day 0 and 2 in heated and non-heated tea (**C**). ^a,b,c^ indicates significant differences *p* < 0.05

**Fig. 5:**
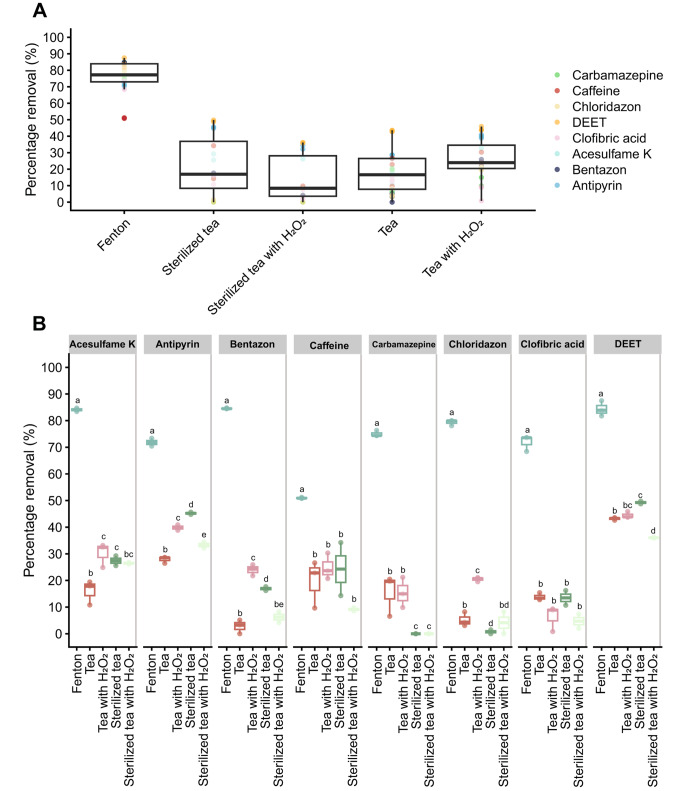
Organic micropollutant removal by a heterologous Fenton-like reaction compared to non- or heat-treated SMS tea with or without addition of extra H_2_O_2_. Overall, the Fenton reaction removed OMPs better than the different tea conditions (**A**). Addition of 0.97 µM H_2_O_2_ to tea had a positive effect on removal of acesulfame K, antipyrine, chloridazon, and clofibric acid (**B**). Untreated tea was the same or better in removal of OMPs except for antipyrine that was better removed by sterilized tea. Extra H_2_O_2_ had 4 out of 8 times a negative effect on removal by sterilized tea. ^a,b,c,d,e^ indicates significant differences *p* < 0.05 (per compound in **B**)

The non-enzymatic activity could be mediated by the Fenton reaction. Chemical analysis of SMS tea showed the presence of 0.03, 0.88, and 0.33 µg L^-1^ of copper, manganese, and iron, respectively (Fig. 4B). These metals can play a role in a heterogeneous Fenton-like reaction (Hussain et al. 2021) that needs H_2_O_2_ as well. Therefore, its concentration in SMS tea was determined. H_2_O_2_ concentration at day 0 was 1.5 and 3.9 µM in untreated tea and heat-treated tea, respectively (Fig. 4C), while these values were 1.5 and 2.9 µM after 2 days of incubation. Similar results were obtained with heat-treated SMS and untreated SMS (data not shown). A solution was made containing Cu^2+^, Fe^3+^, Mn^2+^ and H_2_O_2_ in the concentrations found in SMS tea, except for 0.97 µM H_2_O_2_. This solution removed > 50% of all the OMPs, which was significantly higher than untreated or sterilized (heat-treated and filtered with a 0.2 μm filter) tea (Fig. 5), even after addition of 0.97 µM extra H_2_O_2_ to these extracts.

Addition of H_2_O_2_ to untreated tea had a positive effect on the removal of acesulfame K (16–30%), antipyrine (28–40%), bentazon (3–24%), and chloridazon (5–20%) (Fig. 5B). This effect was not observed in sterilized tea, implying that the H_2_O_2_ promoted enzymes (being secreted or associated to non-filtered microbes) and not a chemical mechanism as the Fenton reaction. Adding H_2_O_2_ had even a negative effect on the removal of antipyrine (45–33%), bentazon (17–6%), and DEET (49–36%) by sterilized tea. This suggests that another mechanism than the Fenton reaction is responsible for removal of these OMPs by sterilized tea. Carbamazepine was only removed by untreated tea, but this mechanism was not stimulated by the addition of extra H_2_O_2_. By contrast, removal of antipyrine and DEET by sterilized tea was 17% and 6% better than untreated tea, respectively. Together, these results indicate four different mechanism to remove OMPs from waste water with SMS tea. They consist of (extra) H_2_O_2_ independent and dependent enzymatic removal, the heterologous Fenton reaction, and another chemical mechanism that is repressed by extra H_2_O_2_.

## Discussion

SMS of a variety of fungi is able to degrade and sorb OMPs (García-Delgado et al. 2017; Hultberg et al. 2020; Leong et al. 2022; Phan and Sabaratnam 2012; Toptas et al. 2014). However, the capacity of SMS of *A. bisporus* to degrade and sorb a mixture of OMPs was not known. Also, the underlying mechanisms of degradation of SMS of fungi have been poorly described (Ghose and Mitra 2022). Here it is shown that SMS of *A. bisporus* and its tea are able to remove a variety of OMPs when present in water. SMS was superior in removal of OMPs when compared to its tea. Highest removal by SMS was observed for carbamazepine with an amount of 68–90% during a up to 7-days incubation, while 40–80% of the other compounds were removed. Carbamazepine was also most efficiently removed by SMS tea in one experiment with a removal of 26%, while bentazon was better removed (with 30%) in another experiment. A total of 0–20% of the other compounds were removed by the tea. To compare, OMPs as acesulfame K (Belton et al. 2020), antipyrine (Durán et al. 2014), bentazon (Gholami et al. 2021), caffeine (Rigueto et al. 2020), carbamazepine (Mohapatra et al. 2014; Zhang et al. 2008), clofibric acid (Sirés et al. 2007) and DEET (Sui et al. 2010) are removed with a 20–100% efficiency by chlorination, ozonation, and / or advanced oxidation processes. However, these studies focused on one compound and / or it was proven to produce toxic residues (Cai et al. 2013). Some OMPs (e.g. acesulfame K, caffeine, clofibric acid, DEET) were described to be removed up to 100% by bacteria in sludge of the WWTPs (Kleinsteuber et al. 2019; Kosjek et al. 2007; Sui et al. 2010), but this took often longer than a week. Batch reactors with *P. ostreatus* SMS were able to remove 95% of endocrine disruptors (e.g. bisphenol A, 17β-estradiol, estriol, 17α-ethinylestradiol, tricolasan, and 4-*n*-nonyphenol) (Křesinová et al. 2018) and 80% of diclofenac (Hultberg et al. 2020), while batch reactors with *Trametes versicolor* removed > 80% of bentazon and diuron (Hu et al. 2021). Teas of mechanically broken *A. bisporus* SMS were able to remove 20–100% of different polyaromatic hydrocarbons (e.g. anthracene, acenapthylene, benzo[*a*]pyrene, benzo[*a*]anthracene, benzo[*ghi*]perylene, dibenzo[*a,h*]antrecene, fluorene, pyrene ) in 24 h (Li et al. 2010). So, removal activity of *A. bisporus* SMS was in the same range compared to other bioremediation experiments. Its tea had a lower removal than described in literature but this may be explained by the fact that in our study we did not prepare tea from mechanically broken SMS, that could release intracellular enzymes. Further, in our study we calculated the degradation at a certain day by subtracting the concentration at day 0. However, it should be noted that the day 0 sample had been incubated for a few hours due to handling time. During this time, degradation is expected to have started (Hussain et al. 2021).

Removal of the OMPs was significantly higher by SMS than its tea. This difference may be explained by the structure of the compounds. For instance, hydrophobicity of compounds can impact enzymatic degradation in solution (Eibes et al. 2007). More hydrophobic compounds would thus be removed better by SMS. However, the more hydrophilic compounds (e.g. acesulfame K and caffeine) were also better removed by SMS than its tea. Another explanation for the higher removal activity of SMS could be that this substrate immobilizes enzymes, increasing their concentration and stability (Cao et al. 1996). Sorption of the OMPs by SMS do not seem to explain the differences between SMS and its tea. Yet, we cannot exclude that we underestimate the sorption. This would be the case when the recovery rate of the sorbed OMPs by the methanol extraction is < 100%, while we assume in our calculations that all OMPs are recovered by the extraction. Studies on spent activated carbonaceous adsorbents have shown recovery rates ranging between 20 and 100% (Oesterle et al. 2020). Yet, these substrates have been developed to strongly adsorb chemicals, which is not the case for SMS. Unfortunately, the recovery rate in the SMS system cannot be determined since we do not have a control that sorbs but does not degrade the OMPs. For instance, non-enzymatic degradation capacity is not affected by heating the SMS and thereby interferes with the recovery rate calculations.

Until now, degradation mechanism of OMPs by fungi have mainly focused on lignin degrading enzymes (Ghose and Mitra 2022). Here we showed that multiple mechanisms play a role in removal of OMPs by SMS and its tea. Heat treatment of SMS and its tea did not reduce the removal of carbamazepine, implying that this compound was not (only) removed by enzymes. In this study, we proposed the possibility of a Fenton-like reaction due to the available metals and H_2_O_2_. A reconstituted Fenton solution based on the concentrations found in tea and SMS removed > 50% of the OMPs, which was significant better than non- or heat-treated tea either or not supplemented with H_2_O_2_. The lower removal in tea could be explained by the presence of anti-oxidants. These molecules can lower the removal rate of the OMPs (Ghahremani-Majd and Dashti 2015). Also, the higher level of dissolved organic matter in the tea could have an negative effect by neutralizing hydroxyl radicals (Lee and Fan 2020). Next to this Fenton reaction, results indicate a non-enzymatic reaction in the sterilized tea that is negatively affected by H_2_O_2_ to remove for example antipyrine, bentazon, and DEET. It is known that removal of pentachlorophenol by the Fenton reaction can be induced by a higher concentration of H_2_O_2_ (Christoforidis et al. 2015). This effect is even higher if humic acids are available in the system. A negative effect on removal by a higher amount of is H_2_O_2_ not described. So, the reason for this negative effect is unknown. Overall, the heat treatment of SMS had a negative effect on removal of OMPs. This suggests that enzymes in SMS are also responsible for removal of OMPs. For 4 out of 8 OMPs addition of H_2_O_2_ to untreated tea even increased this removal, and shows a H_2_O_2_-dependent mechanism of OMP removing enzymes (secreted or present in non-filtered microbes). Probably, these enzymes consist of the lignin degrading enzymes such as manganese peroxidase (Pontes et al. 2018). However, the other 4 OMPs were not better removed after adding H_2_O_2_ to untreated tea. This suggests that H_2_O_2_-independent enzymes may also play a role. These could be the laccases that oxidize the substrates, but also lowly specific monooxygenases such as cytochrome P450 enzymes, flavin-containing monooxygenases, and dioxygenases such as Rieske-type non-heme-iron dioxygenases are known to hydroxylate substrates without using H_2_O_2_ (Anzenbacher and Anzenbacherova 2001; Doddapaneni et al. 2013; Kennes-Veiga et al. 2021). Next to this, demethylation, deamination, and conjugation can be used to modify OMPs in a H_2_O_2_-independent way. Together, this study shows that *A. bisporus* SMS and its tea provide multiple mechanisms to remove a variety of OMPs from waste water. The removal rates are comparable to other reports that used SMS of mushrooms. The fact that SMS of *A. bisporus* is highly available makes this a very promising substrate for bioremediation.

## Electronic supplementary material

Below is the link to the electronic supplementary material.

Supplementary Material 1

## Data Availability

The authors declare that the data supporting the findings of this study are available within the paper. Should any raw data files be needed in another format they are available from the corresponding author upon reasonable request.
