# Supplementary material for: Enzymatic and non-enzymatic removal of organic micropollutants with spent mushroom substrate of Agaricus bisporus
Source: Appl Microbiol Biotechnol. 2024 Apr 19;108(1):301. doi: 10.1007/s00253-024-13132-3 (PMC11031484; doi:10.1007/s00253-024-13132-3)
Supplement: Supplementary file 1 — Supplementary Material 1 [file 253_2024_13132_MOESM1_ESM.pdf]

Supplementary Material

Applied Microbiology and Biotechnology

Enzymatic and non-enzymatic removal of organic micropollutants with spent mushroom substrate of *Agaricus bisporus*

Brigit van Brenk[1], Fleur E.L. Kleijburg[1], Antoine J.B. Kemperman[2], Walter G.J. van der Meer[2,3], Han A.B. Wösten[1*]

[1]Microbiology, Department of Biology, Utrecht University, Padualaan 8, 3584 CH Utrecht, the Netherlands; [2]Membrane Science and Technology cluster, University of Twente, P.O. Box 217, 7500 AE, Enschede, the Netherlands; [3]Oasen, PO BOX 122, 2800 AC, Gouda, the Netherlands

*** Corresponding author**

Prof Dr HAB Wösten

Microbiology, Utrecht University

Padualaan 8, 3584 CH Utrecht, The Netherlands

Telephone: 0031 30 2533448 E-mail: h.a.b.wosten@uu.nl

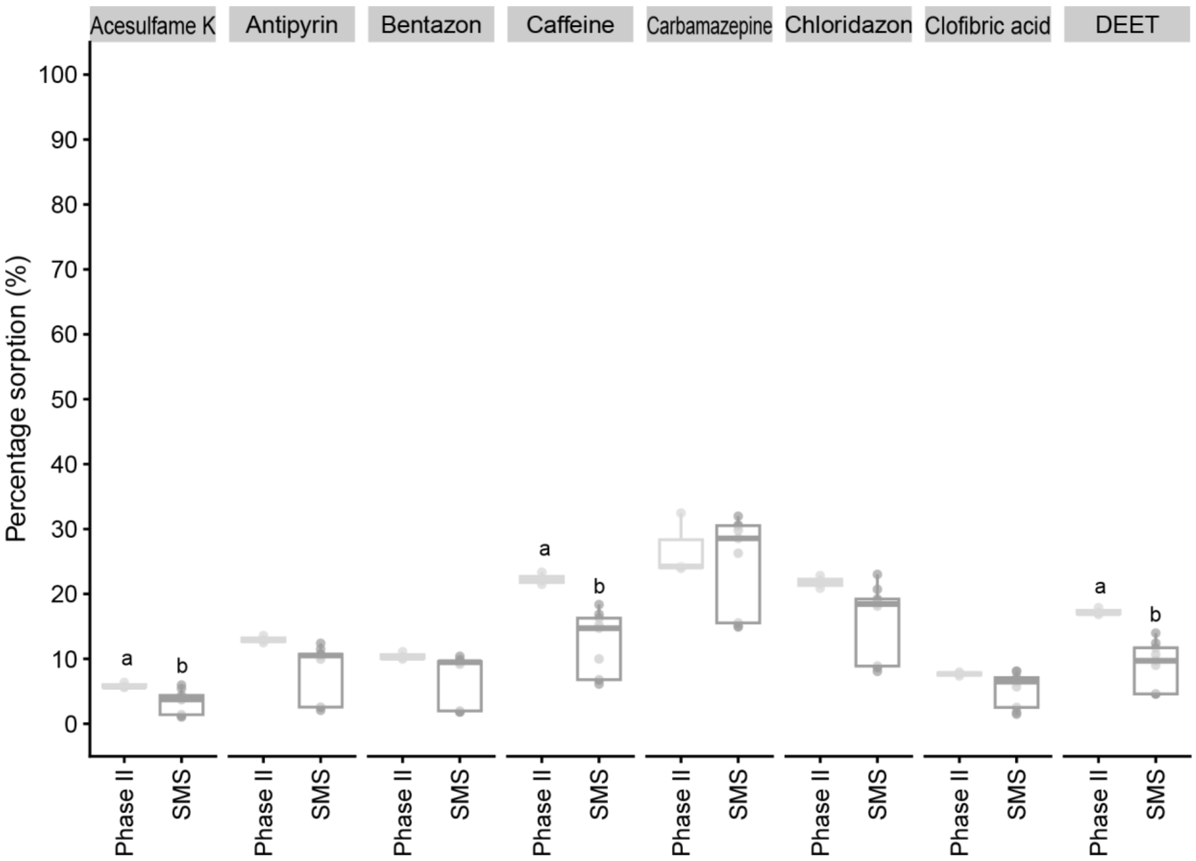

Fig S1. Percentage sorbed OMPs after 2 days of incubation with phase II compost (without *Agaricus bisporus*) or spent mushroom substrate (SMS). [a,b,c] indicates significant differences $p < 0.05$.
